# Incidence, risk factors, and impact on hospital mortality of status epilepticus after subdural hemorrhage in the United States

**DOI:** 10.1186/cc9750

**Published:** 2011-03-11

**Authors:** A Seifi, J Urtecho, M Maltenfort, M Vibbert, W McBride, M Moussouttas, J Jallo, R Bell, F Rincon

**Affiliations:** 1 Thomas Jefferson University, Philadelphia, PA, USA

## Introduction

Patients with intracranial hemorrhages are at risk of seizure activity. Small cohort studies have shown that patients with subdural hemorrhages (SDH) may be at risk of developing status epilepticus (SE). In this study, we sought to determine the epidemiology of SE, the prevalence of risk factors, and the impact on hospital mortality in SDH, using a large administrative dataset.

## Methods

Data were derived from the National Inpatient Sample from 1988 through 2008. We searched for admissions with a primary diagnosis of SDH, and SE. Definitions were based on the International Classification of Diseases, 9th Revision. Adjusted incidence rates, prevalence odds ratios (ORs), and 95% confidence intervals (CIs) were calculated.

## Results

Over the 20-year period, we identified 890,153 admissions with primary diagnosis of SDH and 3,214 of SE. The population-adjusted rate of SDH increased from 9/100,000/year in 1988 to 22/100,000/year in 2008, and similarly, the adjusted rate of SE in SDH increased from 0.05/100,000/year in 1988 to 0.11/100,000/year in 2008. In SDH patients, the risk of SE was higher in older than younger patients (OR, 0.99; 95% CI, 0.99 to 1.0, *P *= 0.06), black than whites (OR, 1.5; 95% CI, 1.2 to 1.9), and in the presence of respiratory failure (OR, 4.3; 95% CI, 3.5 to 5.3), metabolic dysfunction (OR, 1.7; 95% CI 1.3 to 2.26), hematologic disorders (OR, 1.7; 95% CI, 1.3 to 2.26), renal failure (OR, 2.4; 95% CI, 2.1 to 3.26), or central nervous system dysfunction (OR, 2.6; 95% CI, 2.1 to 3.26). The total in-hospital mortality fell from 17% in 1988 to 11% in 2008, yet the number of deaths increased over the study period. In-hospital mortality was higher among SE (OR, 1.6; 95% CI, 1.3 to 2.0) older patients (OR, 1.01; 95% CI, 1.01 to 1.01), women (OR, 1.1; 95% CI, 1.01 to 1.1); and in those with respiratory organ dysfunction (OR, 4.9; 95% CI, 4.7 to 5.2), cardiovascular dysfunction (OR. 2.9; 95% CI, 2.7 to 3.2), hematologic dysfunction (OR, 2.2; 95% CI, 2.1 to 2.3), metabolic dysfunction (OR, 2.5; 95% CI, 2.2 to 2.8), renal dysfunction (OR, 2.0; 95% CI, 1.9 to 2.1).

## Conclusions

Our study demonstrates that the incidence of SDH and SE in these patients is increasing in the United States. The risk of SE was higher among older patients, blacks, and in those with respiratory, metabolic, hematological, and renal system dysfunction. Despite a decline in overall SDH-related mortality, SE increased the risk of in-hospital death.

## References

[B1] Rubin G Epilepsy in chronic subdural hematoma Acta Neurochir 1993 123 39 42 10.1007/BF01476283 8213276

